# Psychosocial impacts of home-schooling on parents and caregivers during the COVID-19 pandemic

**DOI:** 10.1186/s12889-022-12532-2

**Published:** 2022-01-17

**Authors:** Alison L. Calear, Sonia McCallum, Alyssa R. Morse, Michelle Banfield, Amelia Gulliver, Nicolas Cherbuin, Louise M. Farrer, Kristen Murray, Rachael M. Rodney Harris, Philip J. Batterham

**Affiliations:** 1 Centre for Mental Health Research, Research School of Population Health, The Australian National University, 63 Eggleston Road, ACT 2601 Acton, Australia; 2 Centre for Research on Ageing, Health and Wellbeing, Research School of Population Health, The Australian National University, Acton, Australia; 3 Research School of Psychology, The Australian National University, Acton, Australia; 4 National Centre for Epidemiology and Population Health, Research School of Population Health, The Australian National University, Acton, Australia; 5 Fenner School of Environment and Society, The Australian National University, Acton, Australia

**Keywords:** COVID-19, Psychological distress, Impairment, Home-schooling, Parents

## Abstract

**Background:**

The COVID-19 pandemic has been highly disruptive, with the closure of schools causing sudden shifts for students, educators and parents/caregivers to remote learning from home (home-schooling). Limited research has focused on home-schooling during the COVID-19 pandemic, with most research to date being descriptive in nature. The aim of the current study was to comprehensively quantify the psychosocial impacts of home-schooling on parents and other caregivers, and identify factors associated with better outcomes.

**Methods:**

A nationally representative sample of 1,296 Australian adults was recruited at the beginning of Australian COVID-19 restrictions in late-March 2020, and followed up every two weeks. Data for the current study were drawn from waves two and three. Surveys assessed psychosocial outcomes of psychological distress, work and social impairment, and wellbeing, as well as a range of home-schooling factors.

**Results:**

Parents and caregivers who were home-schooling during the COVID-19 pandemic experienced significantly higher levels of psychological distress and work/social impairment compared to those who were not home-schooling or had no school-aged children. A current mental health diagnosis or lower levels of perceived support from their child’s school negatively affected levels of psychological distress, work and social impairment, and wellbeing in parents and caregivers involved in home-schooling.

**Conclusions:**

The mental health impacts of home-schooling were high and may rise as periods of home-schooling increase in frequency and duration. Recognising and acknowledging the challenges of home-schooling is important, and should be included in psychosocial assessments of wellbeing during periods of school closure. Emotional and instrumental support is needed for those involved in home-schooling, as perceived levels of support is associated with improved outcomes. Proactive planning by schools to support parents may promote better outcomes and improved home-schooling experiences for students.

## Background

By the end of March 2020, many countries had implemented strict physical distancing policies, including large-scale or national closure of schools, to reduce the transmission of COVID-19 [1]. According to the United Nations Educational Scientific and Cultural Organisation (UNESCO), by the beginning of April 2020 an estimated 172 countries had instituted nation-wide school closures affecting over 1.4 billion learners [2]. In response, educators had to adapt curriculum and implement new modes of delivery to enable students to participate in remote learning from home (hereafter termed home-schooling), while parents and other caregivers had to manage the supervision of home-schooling alongside their other professional, personal, and parenting roles [3, 4]. In Australia, schools closed nationally to the majority of students at the end of March 2020, with select schools remaining open for vulnerable children, based on young age, social disadvantage, or specific needs [5], and those whose parents or caregivers were healthcare or other essential frontline workers.

 For many parents and caregivers, home-schooling has placed considerable demands on time. It has often required them to balance multiple competing and unfamiliar roles without the usual support of grandparents, or other extended family, friends or teachers [3, 6]. The challenges of home-schooling may be exacerbated by pressure to continue to work from home to keep jobs and businesses running [6]. As a result, some parents and caregivers have had to work longer hours each day to meet work and home-schooling obligations, potentially affecting sleep and reducing time for leisure activities [3, 7].

The availability of resources for schools and families, such as electronic devices and adequate internet service, has also likely impacted home-schooling experiences. Carers of younger school-aged children or those with additional needs may have been particularly affected, as these children typically require closer supervision to complete home-schooling activities. A study in Hong Kong that surveyed parents about their experiences of home-schooling reported that only 14% of primary school students could complete activities without assistance [4].

School closures have been a highly disruptive element of the COVID-19 pandemic, altering the day-to-day lives of children and families. The sudden shift to home-schooling and the challenges it has presented based on factors such as the age and ability of their child(ren), parental income, living conditions (crowded housing or homelessness), and available additional support has placed added pressure on parents and caregivers [3, 8]. In addition, the impact of home-schooling has not been evenly distributed, with caregivers of children with disabilities and diverse educational needs facing higher rates of stress and mental health problems [7, 9]. In turn, increased parental stress may have negatively affected their mental health, the parent-child relationship, and the emotional wellbeing of the child [3, 4]. These factors may have also impacted educational attainment [5].

Given the ongoing COVID-19 lockdowns internationally, and the potential for future pandemics and other system shocks (e.g., fires, floods, earthquakes), there is a clear need to comprehensively quantify the psychosocial impacts of home-schooling on parents and other caregivers, and to investigate individual and environmental characteristics that exacerbate them. Therefore, the current study aimed to (1) assess the impact of home-schooling on parent/caregiver psychological distress, work and social impairment, and wellbeing; and (2) identify factors associated with psychological distress, work and social impairment, and wellbeing among those engaged in home-schooling.

## Methods

### Participants and procedure

The Australian National COVID-19 Mental Health, Behaviour and Risk Communication (COVID-MHBRC) survey was established to longitudinally assess the impact of the COVID-19 pandemic on a representative sample of Australian adults aged 18 years and over [10]. The study consisted of seven waves of data collection, which were completed online on a fortnightly basis and administered through Qualtrics Research Services. Participants were emailed an invitation to complete each survey and were provided a one-week window in which to complete it. Participants received up to five reminders to complete a survey during the week of data collection. Quota sampling was used to obtain a sample of the Australian population from market research panels that was representative on the bases of age group, gender and State/Territory of residence. Written informed consent was obtained from all participants prior to participation in the study. The current study was approved by The Australian National University Human Research Ethics Committee (protocol 2020/152) and the full study protocol is available online (https://psychology.anu.edu.au/files/COVID_MHBRCS_protocol.pdf).

The first wave of data collection commenced on the 28th March 2020 (*N*=1296). Besides demographics and background variables (collected in Wave 1), data for the current study were drawn from waves two (home-schooling variables) and three (mental health outcomes) collected between the 11th and 30th April 2020. Over 73% of the initial sample was retained at Wave 2 (W2; *N*=969). Attrition across subsequent waves was lower, consistently retaining over 90% wave-on-wave (*N* W3=952, W4=910, W5=874; W6=820; W7=762).

### Measures

#### Psychological Distress

The five-item Distress Questionnaire-5 (DQ5; [11]) was measured at wave 3 and used to assess psychological distress over the past two weeks. Items were rated on a 5-point scale ranging from 1 (Never) to 5 (Always). Total scale scores ranged from 5 to 25, with higher scores indicating greater psychological distress. The scale had very good internal consistency in the current study sample (α = 0.93).

#### Work and social impairment

The extent to which work and social activities were impaired by COVID-19 was measured at wave 3 using the Work and Social Adjustment Scale (WSAS; [12]). Participants were asked to rate the level of impairment COVID-19 had caused for five work and social domains (ability to work, home management, social leisure activities, private leisure activities, and ability to form and maintain close relationships) on a 9-point scale ranging from 0 (Not at all impaired) to 8 (Very severely impaired). Total scores on this scale ranged from 0 to 40, with higher scores indicative of greater work and social impairment as a result of COVID-19. The WSAS had very good internal consistency in the current study sample (α = 0.77).

#### Wellbeing

Subjective wellbeing during the past two weeks was assessed at wave 3 using the 5-item World Health Organization Wellbeing Index (WHO-5; [13]). Items were responded to on a 6-point scale ranging from 0 (At no time) to 5 (All of the time), multiplied by four to obtain total scale scores ranging between 0 and 100. Higher scores are indicative of greater wellbeing. The scale had very good internal consistency (α = 0.93).

#### Home-schooling factors

A range of factors associated with home-schooling were also assessed at wave 2 among respondents who reported home-schooling their children due to COVID-19. Respondents to these items could include parents, grandparents, or other caregivers, and included items on the school level of children (primary school/ secondary school), working from home (yes/no), sharing of home-schooling duties (yes/no), and perceived impact on work/daily activity (4-point scale from ‘not at all’ to ‘a lot’). The *amount of support* received from the school was also collated (e.g., online social interactions with teachers and/or peers; real-time lessons; pre-recorded teacher instruction videos; structured activities; list of optional activities; connected with other parents), with total scores on this item ranging from 0 to 6. The *perceived support* received from the school was assessed based on perceptions of school flexibility (e.g., advice from the school to do what best suits each family), how the school facilitated connection to peers, whether the school helped families to enjoy home-schooling, or whether the school caused parents to feel stress or worry about home-schooling. These four items were assessed on a 5-point scale ranging from 0 (Not at all) to 4 (Extremely) and could range from 0 to 16 with higher scores indicating greater perceived support.

#### Demographic and background variables

At wave 1 participants also provided details on their age, gender, and level of educational attainment (Secondary school, certificate/diploma, Bachelor’s degree, higher degree [e.g., PhD]). Participants were also asked if they had ever been diagnosed (past/current) by a clinician (e.g., general practitioner, psychologist, psychiatrist) with anxiety, depression, bipolar disorder, schizophrenia, post-traumatic stress disorder, autism spectrum disorder, alcohol or substance use disorder, eating disorder, or other mental disorder (specify). For the purposes of the current study, these items were combined into a single variable assessing mental health diagnosis history (none/past/current).

##### Statistical analysis

 Between-subject ANOVAs were conducted to compare participants who were home-schooling, with those who had children but were not home-schooling them (‘not home-schooling’) and those who did not have school-aged children, on the key psychosocial outcomes of (i) psychological distress, (ii) work and social impairment, and (iii) wellbeing. A series of linear regression analyses were conducted to identify if demographic, background and home-schooling variables collected at wave 2 were associated with higher levels of psychological distress and work and social impairment, and lower levels of wellbeing measured at wave 3.

## Results

### Impact on psychosocial outcomes

Table 1 presents participant characteristics according to home-schooling status. For demographic factors, participants who reported home-schooling their children were significantly younger and more likely to have a Bachelor’s degree than participants without school-aged children or those not home-schooling their children. There were no significant differences between the three groups in terms of gender or mental health diagnosis.

**Table 1:** Participant characteristics according to home-schooling status

	No school-aged children (*n*=606)		Home-schooling children (*n*=180)		Not home-schooling children (*n*=85)			
	**Mean**	***SD***	**Mean**	***SD***	**Mean**	***SD***	***F***	***p***
**Psychological distress (DQ5)**	10.01	4.88	11.54	5.33	9.82	5.29	6.90	**0.001**
**Work/social impairment (WSAS)**	12.89	9.13	16.25	9.83	12.12	8.01	10.40	**<0.001**
**Wellbeing (WHO-5)**	52.04	25.08	50.36	23.98	51.53	26.74	0.32	0.730
**Age**	50.79	17.46	42.51	10.31	56.88	13.46	28.64	**<0.001**
	***n***	**%**	***n***	**%**	***n***	**%**	***χ2***	***p***
**Mental health diagnosis**							1.61	0.808
Past	112	18.5%	29	16.1%	12	14.1%		
Current	143	23.6%	44	24.4%	19	22.4%		
Never	351	57.9%	107	59.4%	54	63.5%		
**Gender**							5.27	0.261
Male	310	51.2%	87	48.3%	48	56.5%		
Female	296	48.8%	92	51.1%	37	43.5%		
Prefer not to answer	0	0.0%	1	0.6%	0	0.0%		
**Educational attainment**							22.74	**0.004**
Secondary School	173	28.5%	35	19.4%	24	28.2%		
Certificate or diploma	219	36.1%	54	30.0%	37	43.5%		
Bachelor’s degree	180	29.7%	82	45.6%	23	27.1%		
Higher degree	30	5.0%	7	3.9%	1	1.2%		
Prefer not to answer	4	0.7%	2	1.1%	0	0.0%		

The impact of home-schooling on psychological distress, work/social impairment and wellbeing at wave 3 is also presented in Table 1. On average, home-schooling participants scored 1.6 points higher (Cohen’s *d* = 0.32) on the DQ5 measure of psychological distress, *F*(2,872)=7.19, *p*= 0.001, and 3.5 points higher (*d* = 0.38) on the WSAS measure of work/social impairment, *F*(2,869)=9.90, *p*<0.001, compared to participants who were not home-schooling. No differences were observed in levels of wellbeing between groups, *F*(2,873)=0.35, *p*= 0.704.

### Factors associated with psychosocial outcomes among participants who home-schooled

Table 2 presents a summary of the home-schooling variables. The majority of home-schooling participants reported having at least one primary-school aged child (65.4%), while just over half reported working from home while home-schooling (53.8%) and/or sharing the home-schooling duties with another adult (51%). A little under half of home-schooling participants perceived home-schooling to have had some or much impact on their work or daily activities. On average, participants received two home-schooling supports from their school (e.g., structured activities).

**Table 2:** Descriptive statistics for home-schooling variables (*N* = 179)

	*n*	%
**School level of children**		
Primary school (or both)	114	63.7
Secondary school	65	36.3
**Working from home**		
Yes	101	56.4
No	78	43.6
**Sharing home-schooling duties**		
Yes	92	51.4
No	87	48.6
**Perceived impact on work/daily activities**		
Somewhat/a lot	89	49.7
Not at all/A little	90	50.3
	**Mean**	***SD***
**Amount of support received from school (count)**	2.15	1.42
**Perceived supportiveness of school**	9.28	2.96

Table 3 presents the results of the linear regression analyses. Higher levels of psychological distress were significantly associated with a current mental health diagnosis, lower levels of educational attainment, greater perceptions that home-schooling was having an impact on work and daily activities, and lower levels of perceived support from their child’s school. Higher levels of work and social impairment were significantly associated with a current mental health diagnosis, male gender, younger age, and lower levels of perceived support from their child’s school. Lastly, lower levels of wellbeing were significantly associated with past and current mental health diagnosis, and lower levels of perceived support from their child’s school.

**Table 3:** Linear regression analyses assessing the association between demographic and home-schooling factors and home-schooling participants’ levels of psychological distress, work/social impairment and wellbeing

	DQ5 Psychological distress (*N* = 179; *R*^*2*^ = 0.29)				WSAS Work/social impairment (*N* = 179; *R*^*2*^ = 0.26)				WHO-5 Wellbeing (*N* = 179; *R*^*2*^ = 0.23)			
	Estimate	*SE*	*t*	*p*	Estimate	*SE*	*t*	*p*	Estimate	*SE*	*t*	*p*
Mental health diagnosis												
Past	1.809	1.038	1.743	0.083	2.768	1.943	1.425	0.156	-11.721	4.883	-2.400	**0.018**
Current	3.897	0.896	4.348	**<0.001**	5.304	1.677	3.163	**0.002**	-17.982	4.215	-4.267	**<0.001**
Never	Reference				Reference				Reference			
Gender												
Female	-1.403	0.766	-1.832	0.069	-4.660	1.433	-3.251	**0.001**	-1.423	3.602	-0.395	0.693
Prefer not to answer	1.708	4.943	0.346	0.730	-7.621	9.250	-0.824	0.411	-21.717	23.246	-0.934	0.352
Male	Reference				Reference				Reference			
Educational attainment												
Certificate/diploma	-1.394	1.082	-1.288	0.200	-1.115	2.025	-0.551	0.583	-5.206	5.089	-1.023	0.308
Bachelor’s degree	-2.078	1.020	-2.038	**0.043**	-0.810	1.908	-0.424	0.672	-1.513	4.796	-0.315	0.753
Higher degree	-4.648	2.067	-2.249	**0.026**	-0.338	3.867	-0.087	0.931	13.357	9.719	1.374	0.171
Prefer not to answer	-2.014	3.546	-0.568	0.571	-5.769	6.635	-0.869	0.386	11.083	16.676	0.665	0.507
Secondary school or less	Reference				Reference				Reference			
Age	-0.072	0.043	-1.685	0.094	-0.258	0.080	-3.229	**0.002**	-0.250	0.201	-1.242	0.216
Working from home (Y/N)	1.061	0.853	1.244	0.215	0.279	1.596	0.175	0.862	-1.643	4.010	-0.410	0.683
Share home-schooling duties (Y/N)	0.585	0.811	0.721	0.472	0.539	1.518	0.355	0.723	-0.048	3.814	-0.013	0.990
Somewhat/a lot impact (vs. none/little)	1.748	0.750	2.331	**0.021**	2.645	1.403	1.885	0.061	1.653	3.526	0.469	0.640
Amount of support received from school	-0.391	0.272	-1.438	0.152	-0.289	0.509	-0.568	0.571	0.570	1.280	0.445	0.657
Perceived support from school	-0.262	0.128	-2.049	**0.042**	-0.671	0.239	-2.805	**0.006**	2.066	0.601	3.435	**0.001**
Primary vs. secondary school student	-0.946	0.872	-1.085	0.280	0.911	1.631	0.559	0.577	1.028	4.099	0.251	0.802
Intercept	17.719	2.684	6.601	<0.001	33.139	5.022	6.598	<0.001	48.773	12.622	3.864	<0.001

## Discussion

To our knowledge, the current study is the first to comprehensively assess and quantify the psychosocial effects of home-schooling on parents and other caregivers during the COVID-19 pandemic. Overall, this study found that parents and caregivers who were home-schooling during the COVID-19 pandemic experienced higher levels of psychological distress and work/social impairment than those who were not home-schooling or had no school-aged children. Among those home-schooling, younger people with less education and a history of mental health problems had higher psychological distress and lower wellbeing. Work/social impairment was additionally associated with being male. Those who perceived home-schooling to have a higher impact on work and daily activities, and those who believed they had lower levels of support from the school, also experienced greater distress and work/social impairments. This key finding highlights the importance of communication between schools and parents in the context of home-schooling during the COVID-19 pandemic. In particular, the need to acknowledge and support the diverse challenges faced when home-schooling, such as enabling flexibility in expectations and activities.

The findings are consistent with qualitative research suggesting that home-schooling puts enormous time demands and pressure on people who are required to fulfil multiple, and sometimes conflicting, roles [3, 4]. For many parents and caregivers, the time needed to undertake home-schooling duties has adversely impacted their ability to work, or led to a reduction or reallocation of work hours, which may have also reduced their ability to engage in home management and leisure activities [3, 7]. The distress, lowered wellbeing, and lack of support felt by parents during this time has likely been amplified by financial concerns, worries about the health risks of COVID-19, and the inability to draw on usual social networks for support, such as grandparents, friends and other family members, due to strict physical distancing restrictions during the pandemic [3, 6]. The available “down-time” during this period may have been significantly reduced for many parents and caregivers and this is reflected in increased psychological distress, and work and social impairment.

The current study also found that the levels of psychological distress, work and social impairment, and wellbeing experienced by participants who were home-schooling during the pandemic was negatively affected by a current or past mental health diagnosis. People experiencing mental health difficulties may already have a reduced capacity to cope with stress and uncertainty [14, 15]. As the COVID-19 pandemic progresses, and in future major crises, this points to a need to identify people who are highly likely to struggle with the additional responsibilities of home-schooling and ensure tailored support is available to minimise distress and maximise educational outcomes for children.

The importance of support is reinforced by the finding that perceived support from the child’s school was consistently related to all three psychosocial outcomes. Participants who reported higher perceived support from their child’s school tended to report lower levels of distress and impairment, and higher levels of wellbeing. This finding is in line with the wider mental health literature that associates social support with better mental health outcomes [16]. Specifically, it points to the importance of providing all schools with the capacity to deliver the required practical and social support, and appropriate resources to parents and caregivers, during enforced periods of home-schooling, with attention paid to factors that may increase vulnerability to distress. Support may include simple recognition of the challenges faced by non-teachers in education delivery, and reassurance that parents’ and caregivers’ efforts to support their children’s learning are “enough.” Further, cooperation and flexibility from workplaces to ensure parents and caregivers, especially those with a history of mental health problems and/or with young children requiring significant learning support, is also likely to reduce distress and perceived impairments.

Higher psychological distress was also associated with lower levels of educational attainment and higher perceived impact of home-schooling on work and daily activities. Parents and caregivers with lower levels of education may have been less confident in their ability to support learning or found it more challenging due to lower literacy or numeracy skills [17]. Higher perceptions of the negative impacts of home-schooling may have led to feelings of being overwhelmed or reduced feelings of control. This risks further entrenching the social disadvantage already prevalent in those with lower levels of education. Higher levels of work and social impairment were also observed in males and younger participants. Males may have been less accustomed to flexible work arrangements [18], as women are often the primary carers of children, or their positions may have been less amenable to home-schooling disruptions and thus they perceived greater impairments to their work and social functioning. Younger parents and caregivers may have been more likely to have younger children, and thus the time requirement and pressure on them to actively participate in remote home learning activities may have been greater and potentially more disruptive.

Recognising and acknowledging the challenges of home-schooling is important, and should be included in psychosocial assessments of wellbeing during periods of school closure. There is a clear need to provide emotional and instrumental support to parents and other caregivers during school closures so that they can manage all roles effectively, and minimise adverse psychosocial effects. Parents and caregivers need access to support from social networks if available [16], and need schools to communicate realistic expectations, provide adequate educational activities, and supportive feedback that accounts for the unequal spread of perceived impact. Similarly, as teachers are the primary point of contact for students during remote learning, they need to be adequately supported during this time so that they can be available to effectively support students and parents. Whilst the unexpected school closures as a result of COVID-19 necessitated a rapid response to educational support materials that may have been less than ideal in some cases, as the pandemic progresses, it is critical to record and act upon lessons learned about activities that facilitate supported and independent learning for children, and provide greater support for parents and caregivers who are not educators and trying to balance work responsibilities. This is particularly the case for parents and caregivers who may face additional struggles, including those with mental health issues or with lower levels of education, that may undermine confidence or ability to home-school [4] and perpetuate social disadvantage.

The current study has several strengths. Firstly, the data were collected at the peak of home-schooling in Australia in a generally representative population sample. Secondly, data were collected over multiple time points, reinforcing the temporal effects of the findings. However, the study also has some limitations. Although the study was designed to be representative of the Australian population, it is likely that under-privileged groups - those with low income, educational attainment, and employment - were not adequately represented and may have been even more affected by home-schooling [19]. We also did not separately consider the impacts of home-schooling on families with multiple children or on those who had children with a disability or diverse educational needs. Time-poor parents may be less inclined to participate in research panels, so the findings may provide a conservative estimate of the impacts of home-schooling on busy parents.

## Conclusions

 In summary, parents and caregivers engaged in home-schooling during the early stages of the COVID-19 pandemic reported higher levels of psychological distress and work and social impairment than their non-home-schooling peers – both those without school-aged children and those with children still in school. People who were younger, male, had a history of mental health difficulties and/or perceived the impacts of home-schooling on work and daily activities to be higher, or the support of schools to be lower, were particularly affected. Understanding the impacts of home-schooling on parents and caregivers is critical, as periods of home-schooling are likely to continue into the future. In addition, the functioning of parents and caregivers can impact upon the parent-child relationship, child wellbeing and potentially the academic outcomes of children during periods of lock-down.

## Data Availability

The data that support the findings of this study are available from the corresponding author on reasonable request.
